# Analysis of tissue inflammatory response, fibroplasia, and foreign body reaction between the polyglactin suture of abdominal aponeurosis in rats and the intraperitoneal implant of polypropylene, polypropylene/polyglecaprone and polyester/porcine collagen meshes

**DOI:** 10.1590/ACB360706

**Published:** 2021-09-03

**Authors:** Waston Gonçalves Ribeiro, Adriana Carneiro Corrêa Nascimento, Larissa Brito Ferreira, Danilo Dallago De Marchi, Gustavo Moraes Rego, Carlos Toshinori Maeda, Gyl Eanes Barros Silva, Ricardo Artigiani, Orlando Jorge Martins Torres, Marcos Bettini Pitombo

**Affiliations:** 1 Fellow PhD degree. Postgraduate Program in Health Sciences - Faculty of Medical Sciences - Universidade do Estado do Rio de Janeiro (UERJ) - Rio de Janeiro (RJ), Brazil.; 2 Resident. General Surgery Residency Program - Hospital Universitário - Universidade Federal do Maranhão (HU-UFMA) - Sao Luis (MA), Brazil.; 3 Graduate student. School of Medicine - Universidade Federal do Maranhão (UFMA) - Sao Luis (MA), Brazil.; 4 Master. Division of Surgical Gastroenterology - Department of Surgery - Universidade Federal de São Paulo (UNIFESP) - Sao Paulo (SP), Brazil.; 5 PhD. Department of Pathology – Hospital Universitário - Universidade Federal do Maranhão (HU-UFMA) - Sao Luis (MA), Brazil.; 6 PhD. Division of Surgical Gastroenterology - Department of Pathology - Universidade Federal de São Paulo (UNIFESP) - Sao Paulo (SP), Brazil.; 7 PhD, Chairman, Full Professor. Department of Surgery - Universidade Federal do Maranhão (UFMA) - Sao Luis (MA), Brazil.; 8 PhD, Associate Professor - Department General Surgery - Faculty of Medical Sciences - Universidade do Estado do Rio de Janeiro (UERJ) - Rio de Janeiro (RJ), Brazil.

**Keywords:** Surgical Mesh, Inflammation, Collagen, Foreign Body Reaction, Rats

## Abstract

**Purpose:**

To compare tissue inflammatory response, foreign body reaction, fibroplasia, and proportion of type I/III collagen between closure of abdominal wall aponeurosis using polyglactin suture and intraperitoneal implant of polypropylene, polypropylene/polyglecaprone, and polyester/porcine collagen meshes to repair defects in the abdominal wall of rats.

**Methods:**

Forty Wistar rats were placed in four groups, ten animals each, for the intraperitoneal implant of polypropylene, polypropylene/polyglecaprone, and polyester/porcine collagen meshes or suture with polyglactin (sham) after creation of defect in the abdominal wall. Twenty-one days later, histological analysis was performed after staining with hematoxylin-eosin and picrosirius red.

**Results:**

The groups with meshes had a higher inflammation score (p < 0.05) and higher number of gigantocytes (p < 0.05) than the sham group, which had a better fibroplasia with a higher proportion of type I/III collagen than the tissue separating meshes (p < 0.05). There were no statistically significant differences between the three groups with meshes.

**Conclusions:**

The intraperitoneal implant of polypropylene/polyglecaprone and polyester/porcine collagen meshes determined a more intense tissue inflammatory response with exuberant foreign body reaction, immature fibroplasia and low tissue proportion of type I/III collagen compared to suture with polyglactin of abdominal aponeurosis. However, there were no significant differences in relation to the polypropylene mesh group.

## Introduction

The tissue’s resistance to tensile strength essentially depends on the protein composition of the extracellular matrix^1^. The quantity and quality of proteins that compose this matrix provide support for the tissue and a habitable environment for cells. The use of a synthetic mesh for the correction of hernias in the abdominal wall aims to provide the extracellular matrix with greater resistance, which is created in the process of incorporating and integrating the mesh into the newly formed tissue^2^^,^ 3. Mesh filaments play a role similar as the one of the structural proteins that make up the matrix, such as the different types of collagen (e.g., type I collagen and type III collagen)^4^. The implantation of a synthetic biomaterial induces an inflammatory tissue response, fibroplasia, and foreign body reaction by the recruitment, proliferation, and cell differentiation accompanied by synthesis and deposition of proteins with a structural function, such as collagen, in varied proportions^4^^-^ 8.

The response of the host tissue to the implantation of a biomaterial, such as meshes that repair abdominal wall hernias, is modulated by the mesh biocompatibility, type of polymer, weight, textile porosity, shape and size of pores, thickness of filaments, and dimensional arrangement of meshes, which may affect the intensity of inflammatory response, fibroplasia, and foreign body reaction responsible for the incorporation of the prosthesis^2^^,^ 3 ^-^ 6. Thus, chemical, physical and biomechanical properties of the implanted mesh may affect the quality, quantity, and proportion of deposition of collagen and other proteins that make up the extracellular matrix inside the pores and on the periphery of filaments that make up the mesh, as well as the invasion, proliferation, and cell differentiation, besides neovascularization^4^^,^ 6 ^,^ 8 ^-^ 9.

Collagen is structurally and functionally the key protein and the main protein component of the extracellular matrix. Although there are more than 20 different types of collagen, the type I and the type III are the most common. They are related to biomechanical resistance of the connective tissue of fascia, aponeuroses, tendons, skin and fibrous tissues^1^. Typically, type I collagen is the most robust and resistant. Known as a mature collagen, it predominates in the late phase of the wound healing process. The type III has less resistance to tensile strength and predominates in the early phase of wound healing^10^. Type I and type III collagen molecules coexist in a same collagen fibril, and the increase in the proportion of type III collagen determines the formation of thinner collagen fibers, whose tensile strength is less resistant. The reduction in the proportion between type I/type III collagen is related to the appearance of primary, secondary and recurrent abdominal wall hernias^11^^,^ 12.

Tissue separating meshes or double-sided meshes have two fundamental purposes. The first concerns the parietal face, commonly macroporous, composed of a synthetic polymer that must be incorporated into the musculoaponeurotic plane, which must determine a greater support for the abdominal wall, reducing the risk of hernial recurrence, chronic pain and foreign body sensation, not harming the wall biomechanics, and respecting its anisotropy^3^^,^ 13. In turn, the mesh’s visceral face is commonly microporous or laminar, made of synthetic or biological polymers and has anti-adhesive, biodegradable or permanent behavior. Its primary purpose is to minimize the appearance of adhesions between intra-abdominal structures and the visceral surface of the mesh. However, this layer must not delay or negatively affect the neoperitonization process of its surface^14^. Secondarily, this interface, mediated by the tissue separating barriers, should compromise neither the inflammation nor the fibroplasia process responsible for the incorporation of the macroporous parietal face of the mesh^15^^-^ 18.

Despite the advance in the development of synthetic and biological meshes to repair hernias in the abdominal wall, the ideal mesh is not yet available, and many challenges remain in relation to absorbable and non-absorbable meshes. There is a need for permanent investigations that identify meshes with combinations of polymers and coatings capable of promoting the mechanical resistance and support of the host tissue, but without implying an exacerbated inflammatory reaction or foreign body reaction, which promotes a fibroplasia process with adequate collagen deposition and a mesh incorporation with a balanced and harmonic mesh-tissue interaction^3^^,^ 8.

The aim of this study was to understand the mesh-tissue interaction by evaluating and analyzing the inflammatory tissue response, foreign body reaction, fibroplasia response, and type I/III collagen proportion between the closure of the musculoaponeurotic plane of abdominal wall using polyglactin suture and the intraperitoneal implant of a polypropylene mesh and two composite meshes made of absorbable, anti-adhesive polypropylene/polyglecaprone and polyester/porcine collagen barriers to repair defects induced in the abdominal wall of Wistar rats.

## Methods

This study complies with the Brazilian legislation for the use of experimental animals (Arouca Law no. 11.794/2008) and the standards of the Brazilian College of Animal Experimentation (COBEA). It was analyzed and approved by the Ethics Committee on the Use of Animals (CEUA) of Universidade Federal do Maranhão (UFMA), registration no. 23115.011726/2016-51.

Forty Wistar rats (*Rattus norvegicus albinus*), adult males, with a mean weight of 307 ± 33 g and 60 days of life, were selected from the Biotherium, UFMA. The animals were kept in a polypropylene cage under constant environmental conditions, receiving a diet for rats and water *ad libitum* for seven days for adaptation. There was noise control. The temperature was 22°C ± 2°C, the relative humidity was between 40 and 60%, and the light/dark cycles were of 12/12 hours.

**Figure 1:**
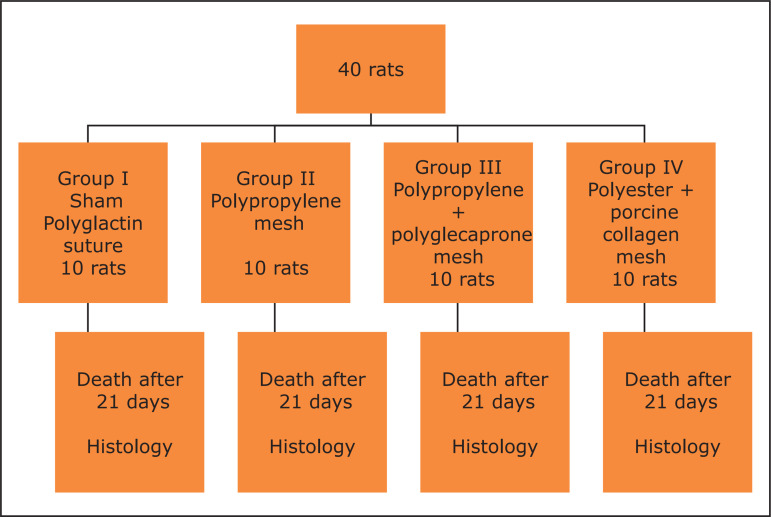
Experimental study that compares the histological findings of the intraperitoneal implant between three different types of mesh (polypropylene, polypropylene/polyglecaprone and polyester/porcine collagen) and the closure of abdominal wall aponeurosis with polyglactin suture (sham group) in Wistar rats.

The rats were randomly assigned to four groups with ten experimental units each (Fig. 1) and subjected to a median laparotomy and an abdominal wall defect repaired with a 4 × 3 cm macroporous meshes implanted intraperitoneally according to the selected group. In Group I without mesh (Sham), the musculoaponeurotic plane closure was performed with a polyglactin 4.0 suture (Novosyn®, B. Braun Surgical SA, Barcelona, Spain); Group II with polypropylene mesh - Optilene® *Mesh* (B. Braun Surgical SA, Barcelona, Spain); Group III with polypropylene mesh with polyglecaprone - Physiomesh® *Flexible Composite Mesh* (Ethicon, Somerville, NJ, USA); and Group IV with a polyester mesh with glycerol and collagen previously hydrated with 0.9% saline solution for one minute - Symbotex® Composite *Mesh* (Covidien, Trévoux, France).

After given an anesthesia with a mixture of xylazine chloride at 2% in a dose of 10 mg/kg and ketamine hydrochloride at 10% in a dose of 100 mg/kg, intramuscularly administered, the animals were submitted to a laparotomy through a medial incision with 4 cm of extension immediately caudal to the xiphoid appendix and to a plane dieresis with dissection between the skin-adipose and musculoaponeurotic planes up to 2 cm on each side of the median line, followed by opening of the abdominal cavity in the alba line measuring 2.5 cm.

One suture point was made using polypropylene 4.0 (Prolene®, Ethicon, Somerville, NJ, United States) on each side of the incision, everting the edges of the abdomen rectum muscle, without covering the peritoneum, thus creating a defect with 2.5 × 1.5 cm (area = 3.75 cm^2^), without any need for abdominal wall resection^19^. According to the allocation, one of the synthetic, lightweight and macroporous meshes with dimensions of 4 × 3 cm (area = 12 cm^2^) was fixed intraperitoneally by six transfixing U suture points in the musculoaponeurotic plane with a polypropylene 4.0 suture (Prolene®, Ethicon, Somerville, NJ, United States) applied in the four corners of the mesh and in the midpoint between the caudal and cranial point on each side. The knots remained in the previously dissected subcutaneous space.

In the sham group, the closure of the musculoaponeurotic plane was performed using a continuous non-anchored suturing with polyglactin 4.0 suture (Novosyn®, B. Braun Surgical S.A., Barcelona, Spain) and a cylindrical needle. In all groups, skin closure was performed with continuous, non-anchored transdermal suturing using a polyglactin 4.0 suture (Novosyn®, B. Braun Surgical S.A., Barcelona, Spain) and a cylindrical needle.

After 21 days, the rats were killed with a mixture of 2% xylazine hydrochloride at a dose of 40 mg/kg and 10% ketamine hydrochloride at a dose of 400 mg/kg, intramuscularly administered. A U-shaped incision was performed involving all anatomical planes of the anterior abdominal wall, bordering the lateral borders of the abdominal wall and groin (lower limit). The flap remained attached only to the costochondral border.

The removed parts were cleaned with 0.9% sodium chloride and placed in a container with 10% buffered formaldehyde. All of them were identified and sent to the Pathological Anatomy Service of the University Hospital (UFMA), where they were prepared and included in paraffin blocks. Three μm thick cuts across the mesh and the polyglactin suturing were obtained in a microtome and stained with hematoxylin-eosin (HE) and picrosirius red, mounted on slides, and covered by coverslips.

**Figure 2:**
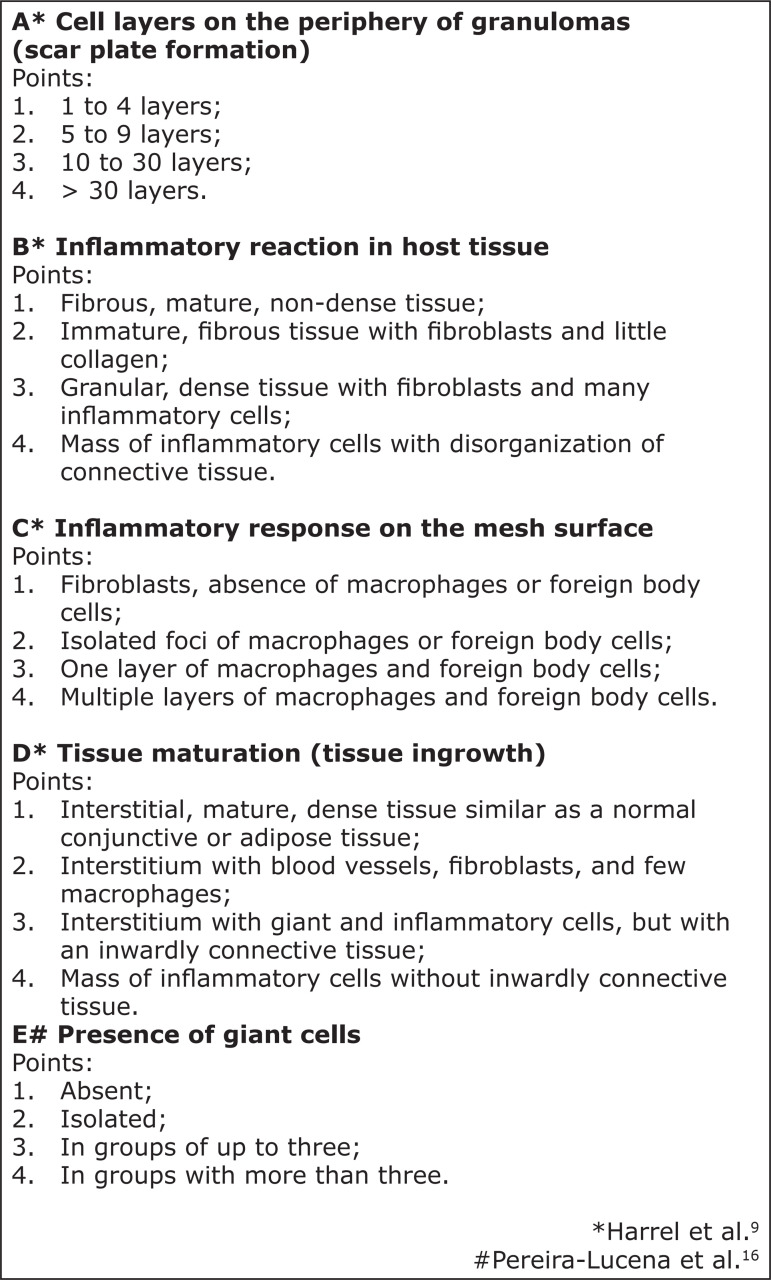
Histological score for assessing tissue inflammatory response and foreign body reaction after experimental mesh implantation to correct abdominal wall defects.

The histological analysis of the inflammatory tissue response was performed at the Department of Pathology of Universidade Federal de São Paulo, by a pathologist with experience on inflammatory responses and fibroplasia after the experimental implantation of synthetic meshes^15^^,^ 16 ^,^ 20 ^,^ 21. The sections stained with HE were examined using an Axio Scope.A1® (Carl Zeiss, Jena, Germany) optical microscope. The intensity and quality of the inflammatory response and the foreign body reaction involving the intraperitoneal mesh implant were evaluated by the tissue inflammation score, as described by Harrell *et al.* 9 and adapted by Pereira-Lucena *et al.* 16, with an objective of 40 (×400) (Fig. 2).

**Figure 3:**
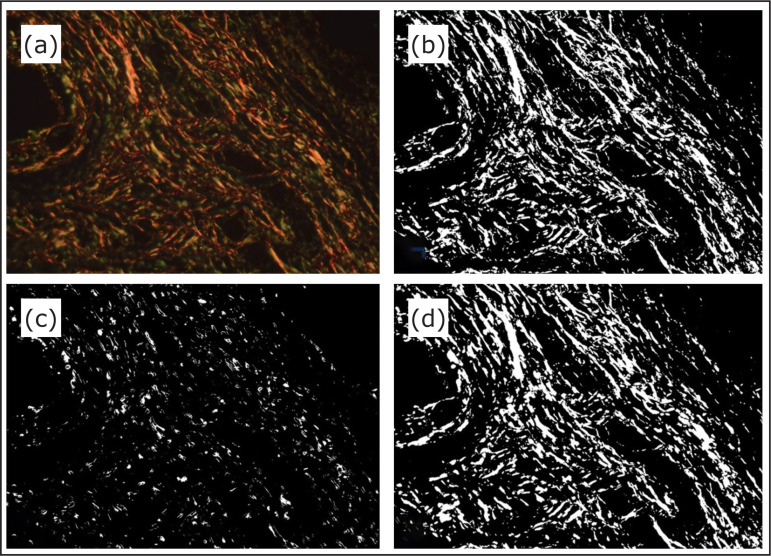
Videomorphometric analysis of collagen using the software ImageJ (version 1.52) in an animal from the polypropylene mesh group: **(a)** Photomicrograph of a sample stained by picrosirius red (x400 magnification); **(b)** Binary image of type I collagen; **(c)** Binary image of type III collagen; **(d)** Binary image of total collagen.

The analysis of fibroplasia response was performed using the videomorphometric technique to assess the sections stained with picrosirius red using an Axio Scope.A1® (Carl Zeiss, Jena, Germany) optical microscope with polarized light associated with an AxioCam MRc® (Carl Zeiss, Jena, Germany) video camera with 1,300 × 1,030 pixels resolution and x400 magnification. The area with the highest concentration of collagen around the filaments of the mesh was chosen. It was characterized by the presence of type I collagen fibers (red or orange color) and type III collagen fibers (green or greenish-yellow color). A Toshiba Tecra A40-D laptop and the software ImageJ version 1.52 free download (public domain) were used for image analysis. The videomorphometry process consisted of quantifying the pixels that make up type I and type III collagen and total collagen after the creation of binary images from the selected photomicrograph of each slide (Fig. 3). The results were expressed by the amount and proportion of type I/III collagen of collagen fibers of each image.

The results are presented in tables and linear dispersion diagram. The analysis of variance was performed by F-test, and the normality test by the Shapiro-Wilk test. For the analysis of quantitative variables, the Kruskal-Wallis test and the *post hoc* multiple comparisons test (Dunn’s test) were used for non-parametric data at a significance level of 5%. The Spearman’s correlation test was used at a significance level of 5%. The software BioEstat®, version 5.3 (AnalystSoft), was used for statistical analysis.

## Results

There was a statistically significant difference between groups as for the number of cell layers on the periphery of granulomas or scar plate (p < 0.0001). It was characterized by the polypropylene mesh group, which showed more layers of cells on the periphery of granulomas compared to the other groups (p < 0.05), both the groups with tissue separating meshes (polypropylene/polyglecaprone and polyester/porcine collagen) and the sham group (Table 1). Approximately 80% of the samples in the polypropylene mesh group had five to nine layers of cells (two points) on the periphery of granulomas. In contrast, in the other groups, one to four layers (one point) predominated.

**Table 1:** Comparison of inflammatory reaction and its variables between the sham (polyglactin suture), polypropylene mesh, polypropylene/polyglecaprone mesh and polyester/porcine collagen mesh groups after staining with hematoxylin-eosin and analysis by optical microscopy.

	Group I Sham Median Min-Max	Group II Polypropylene Median Min-Max	Group III Polypropylene/ polyglecaprone Median Min-Max	Group IV Polyester collagen Median Min-Max	Kruskal-Wallis test (p-value) Dunn’s test (p-value)
A. Cell layers on the periphery of granulomas					p < 0.0001
	1	2	1	1	I × II (p < 0.05)
	1-1	1-2	1-2	1-2	II × III (p < 0.05)
	*	*	*	*	II × IV (p < 0.05)
B. Inflammatory reaction in host tissue	1	2	3	3	p < 0.0001
	1-1	2-3	2-4	2-4	I × III (p < 0.05)
	*		*	*	I × IV (p < 0.05)
C. Inflammatory response on the mesh surface					p < 0.0001
	1	4	4	3	I × II (p < 0.05)
	1-1	3-4	3-4	3-4	I × III (p < 0.05)
	*	*	*	*	I × IV (p < 0.05)
D. Tissue maturation	1	2	3	3	p < 0.0001
	1-1	2-3	3-4	2-4	I × III (p < 0.05)
	*		*	*	I × IV (p < 0.05)
E. Presence of giant cells					p < 0.0001
	1	3	4	3	I × II (p < 0.05)
	1-1	2-4	4-4	3-4	I × III (p < 0.05)
	*	*	*	*	I × IV (p < 0.05)
Total inflammation score					p < 0.0001
	5	13	15	13	I × II (p < 0.05)
	5-5	10-15	14-18	12-18	I × III (p < 0.05)
	*	*	*	*	I × IV (p < 0.05)

Min: minimum; max; maximum; Kruskal-Wallis test + *post hoc* multiple comparisons test (Dunn’s test) + 5% bilateral significance level (p < 0.05); *

* groups that showed statistically significant differences towards the analyzed variable.

**Figure 4:**
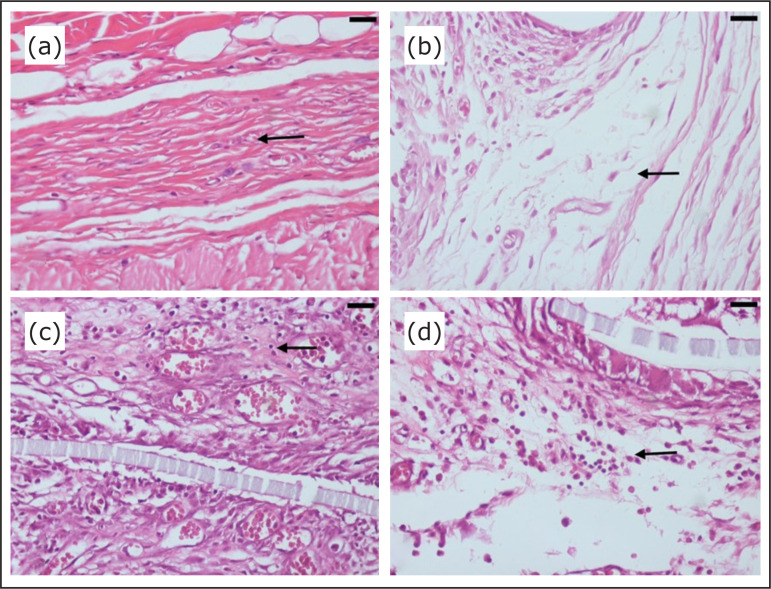
Photomicrographs of the inflammatory reaction in the host tissue stained with hematoxylin-eosin: **(a)** Fibrous, mature tissue in the sham group (polyglactin suture); **(b)** Immature, fibrous tissue with fibroblasts and little collagen in the polypropylene mesh group; **(c)** Granular, dense tissue with fibroblasts and many inflammatory cells in the polypropylene/polyglecaprone mesh group; **(d)** Inflammatory cell mass with disorganization of the connective tissue in the polypropylene/polyglecaprone mesh group (magnification: x400; arrows: area of interest; bar: 50 μm).

Regarding the inflammatory reaction in the host tissue, there was a statistically significant difference between the groups (p < 0.0001), especially between the sham group and the tissue separating meshes (polypropylene/polyglecaprone and polyester/porcine collagen) ones (p < 0.05), although there was no significant difference between the sham group and the group with mesh made exclusively with polypropylene. This difference was characterized by a minimal inflammatory tissue reaction in the sham group and a moderate one in the polypropylene mesh group. In contrast, in groups with tissue separating meshes, there was a more intense tissue inflammatory reaction with disorganized and immature fibroplasia. However, there was no significant difference in relation to the polypropylene mesh group (Fig. 4).

There was a statistically significant difference between the groups as for inflammatory response on the mesh surface (p < 0.0001), with emphasis on the sham group, in contrast to the other groups with meshes (p < 0.05), notably in comparison to the groups with tissue separating meshes (polypropylene/polyglecaprone and polyester/porcine collagen), which were characterized by a greater number of macrophages and giant cells. However, there were no significant differences between groups with polypropylene, polypropylene/polyglecaprone and polyester/porcine collagen meshes.

Regarding tissue maturation, there was a statistically significant difference between the groups (p < 0.0001). The sham group showed a greater tissue maturation and was statistically significant compared to the groups with tissue separating meshes (polypropylene/polyglecaprone and polyester/porcine collagen) (p < 0.05). Although there was a tendency for a better tissue maturation compared to the polypropylene mesh, there was no significant difference between the sham group and the polypropylene mesh group. Similarly, there were no statistically significant differences between the groups with meshes. However, the polypropylene mesh group showed a tendency towards a greater tissue maturation in comparison with tissue separating meshes (polypropylene/polyglecaprone and polyester/porcine collagen).

**Figure 5:**
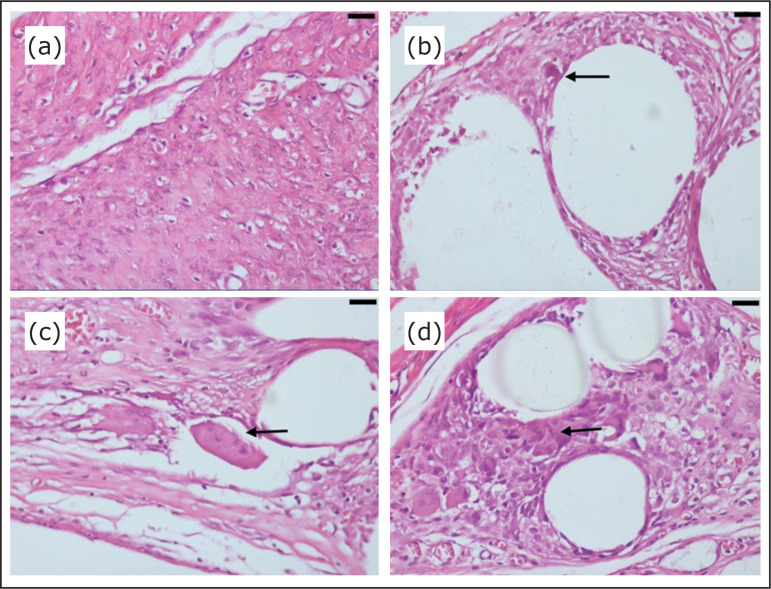
Photomicrographs of giant foreign body cells stained with hematoxylin-eosin: **(a)** Absent in the sham group (polyglactin suture); **(b)** Isolated in the polypropylene mesh group; **(c)** Group of up to three cells in the polyester/porcine collagen mesh group; **(d)** Groups with more than three giant cells in the polyester/porcine collagen mesh group (magnification: x400; arrows: giant cells; bar: 50 μm).

Regarding the number of giant cells, there was a statistically significant difference between the groups (p < 0.0001). This difference occurred between the sham group and all groups with meshes (p < 0.05). There were no statistically significant differences as for the number of gigantocytes between groups with meshes regardless of the presence or absence of the anti-adhesive barriers. The sham group showed absence of gigantocytes, while the polypropylene/polyglecaprone mesh group was uniform with groups of giant cells above three in all animals (Fig. 5).

With reference to the total inflammation score, there was a statistically significant difference between the groups (p < 0.0001). This difference occurred between the sham group and all groups with meshes (p < 0.05). However, there were no statistically significant differences in relation to the total sum of inflammation scores between the groups with meshes regardless of the presence or absence of the anti-adhesive barriers. In the groups with meshes, the lowest values for inflammation score occurred in the polypropylene mesh group, with a tendency for higher values in groups with tissue separating meshes.

**Figure 6:**
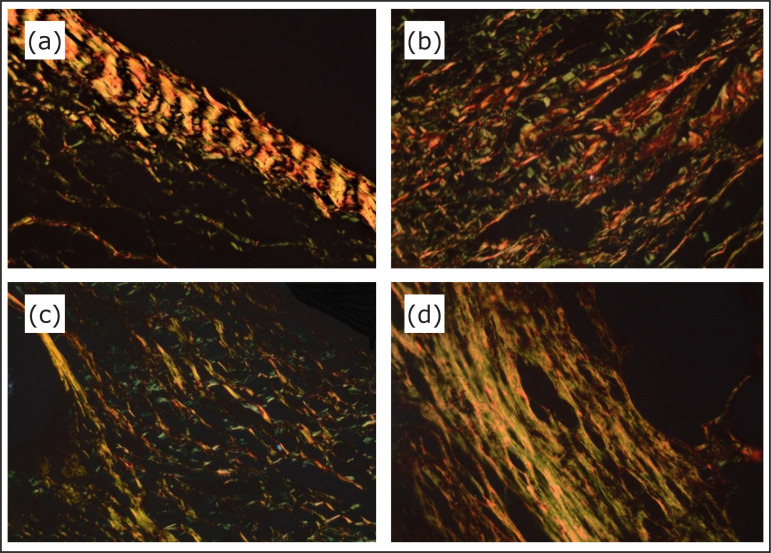
Photomicrographs of type I and type III collagen analysis after staining with picrosirius red: **(a)** Group I – polyglactin suture, **(b)** Group II – polypropylene mesh, **(c)** Group III – polypropylene/polyglecaprone mesh, **(d)** Group IV – polyester/porcine collagen mesh (magnification: x400).

About the proportion of type I/III collagen between the mesh and the host tissue, there was a statistically significant difference between the groups (p = 0.0015). This difference occurred between the sham group and tissue separating meshes (polypropylene/polyglecaprone and polyester/porcine collagen) (p < 0.05). The sham group had a higher proportion of type I/III collagen, but there was no significant difference between the sham group and the polypropylene mesh group as for the proportion of type I/III collagen. Likewise, there were no statistically significant differences between the three groups with meshes. However, the polypropylene/polyglecaprone mesh group had the lowest mean in the proportion of type I/III collagen compared to the polypropylene and polyester/porcine collagen meshes groups, while the polypropylene mesh group had the highest mean among all groups with meshes. Microscopic analysis of slides in the sham group stained with picrosirius red showed that the type I collagen fibers had a more uniform distribution, grouped in dense and birefringent bundles, in relation to all other groups with meshes. Among groups with meshes, the polypropylene mesh group showed the best organization and distribution of type I collagen fibers (Fig. 6).

**Figure 7:**
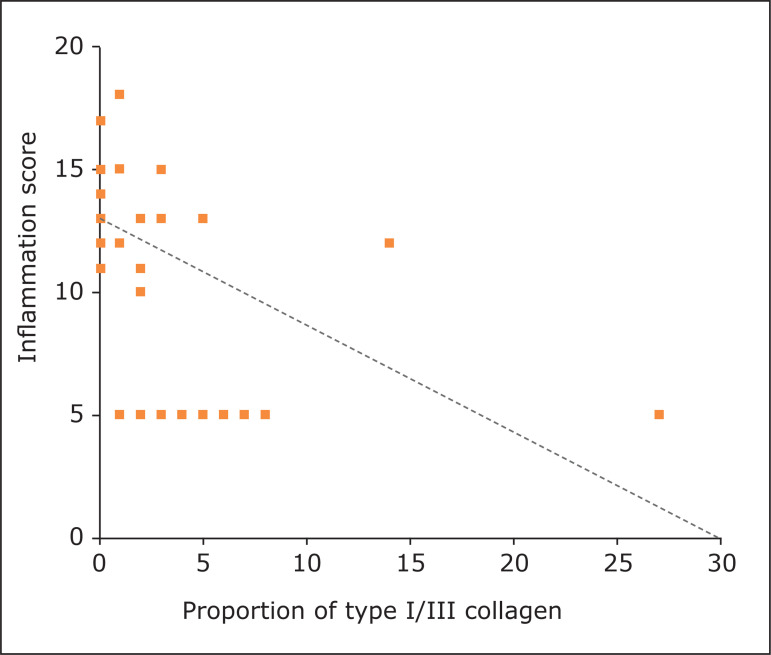
Spearman correlation coefficient (-0.69) and p < 0.0001 between the tissue inflammation score and the proportion of type I/III collagen in the sham group (polyglactin suture) and all groups with meshes (polypropylene, polypropylene/polyglecaprone, and polyester/porcine collagen).

There was a negative correlation between the inflammation score and the proportion of type I/III collagen, with statistical significance, negative Spearman correlation coefficient (-0.69) and p < 0.0001 (Fig. 7).

## Discussion

The implantation of an intraperitoneal synthetic mesh to correct abdominal wall defects determines a host tissue response characterized by an acute inflammatory reaction accompanied by fibroplasia, which is marked by the deposition of various types of collagen in the newly formed extracellular matrix, especially type I and type III collagen, in varying proportions and a foreign body reaction characterized by the presence of giant cells or gigantocytes around the mesh filaments and their absorbable and non-absorbable components^4^^-^ 7. The reciprocal interaction between the mesh and the tissue, which occurs during the process incorporating the implanted mesh, can affect the biomechanical properties and the host tissue of the meshes. Therefore, it may directly impact the results and the performance of meshes used to repair hernias of the abdominal wall^2^^,^ 8.

In the present study, the intraperitoneal implantation of tissue separating meshes using polypropylene/polyglecaprone and polyester/porcine collagen associated with a more intense and durable tissue inflammatory response; a immature fibroplasia response, characterized by a lower tissue proportion of type I/III collagen; a foreign body reaction marked by a greater amount of macrophages and giant foreign body cells compared to repair with polyglactin suture in the musculoaponeurotic plane.

The sham group showed the lowest inflammatory response in the host tissue, which was statistically significant in relation to the groups with polypropylene/polyglecaprone and polyester/porcine collagen meshes, although with no significant difference towards the group with polypropylene mesh. This difference was characterized by a minimal and uniform tissue inflammatory response in the sham group, demonstrated by the presence of fibrous and mature tissue and a reduced inflammatory cellularity. Although there was no significant difference between the sham group and the polypropylene mesh group, the latter showed a moderate tissue inflammatory response characterized by the presence of immature fibrous tissue, with fibroblasts and little collagen. In contrast, the groups with polypropylene/polyglecaprone and polyester/porcine collagen meshes outlined a more intense tissue inflammatory response marked by the presence of a greater number of inflammatory cells, granularity, and disorganized fibroplasia, predominantly in the polyester/porcine collagen mesh group. However, there were no statistically significant differences between the three groups with meshes in relation to the inflammatory response in the host tissue.

Pereira-Lucena *et al*.^16^ conducted an experimental study with rats implanted with three different types of synthetic mesh: microporous heavy polypropylene, light polypropylene/polyglactin, and light polypropylene/titanium. They evaluated the tissue inflammatory response and early and late fibroplasia associated with immunohistochemical analysis with antibodies against pro-inflammatory molecules. The authors concluded that the presence of absorbable material in tissue separating meshes can potentiate the synthesis of pro-inflammatory mediators, which determine a more intense and longer inflammatory response and impair collagen deposition and maturation, compromising the performance of prosthesis by interfering with the fibroplasia process and the incorporation of the implanted mesh. However, there was no statistically significant difference in relation to the systemic inflammatory response between the groups after analysis of pro-inflammatory serum cytokines^15^.

Pascual *et al*.^22^ carried out an experimental study with rabbits and implanted different types of polypropylene meshes, including a partially absorbable mixed mesh made of polypropylene and polyglecaprone, and concluded that the use of absorbable material in fiber conformations and mesh textures could increase the production of inflammatory mediators, which could in turn contribute to a more pronounced acute inflammatory response with a higher percentage of macrophages.

These conclusions corroborate and create a reasonable explanation for a greater and significant inflammatory tissue response as identified in the groups with tissue separating meshes compared to the sham group of the present study. However, despite the tendency towards a greater inflammatory response in the polypropylene/polyglecaprone and polyester/porcine collagen meshes groups, there were no significant differences in relation to the polypropylene mesh group.

In contrast to the differences observed in inflammatory tissue response between the sham group and the tissue separating meshes groups of the present study and the findings described by Pereira-Lucena *et al*.^15^, Garcia *et al*.^18^ carried out an experimental study with rabbits with intraperitoneal implants of two different types of mesh to correct a defect induced in the abdominal wall, a lightweight and macroporous polypropylene mesh and the second type was a double-sided mesh composed of light-weight and macroporous polypropylene partially coated with polymerized and purified type I bovine collagen. The tissue separating mesh significantly reduced adhesions to intra-abdominal viscera, but there were no significant differences between the groups as for the degree of acute or chronic inflammation and foreign body reaction.

The tissue maturation evaluated by the analysis of the slides stained with HE was better in the sham group and statistically significant compared to the groups composed of polypropylene/polyglecaprone and polyester/porcine collagen meshes, although without significant differences in relation to the polypropylene mesh group. This gradient of tissue maturation was characterized by the presence of mature, dense interstitial tissue, similar as the normal conjunctive or adipose tissue. In turn, the polypropylene mesh group, although showing a tendency towards a better tissue maturation, did not show statistically significant differences in relation to the groups with polypropylene/polyglecaprone and polyester/porcine collagen meshes, which also did not present any significant differences between each other.

Maeda *et al*.^20^, in a study with intraperitoneal implantation of polypropylene mesh with and without absorbable polydioxanone anti-adhesive barrier, concluded that a lightweight and macroporous polypropylene mesh favors an early more intense inflammatory reaction than the heavy-weight and microporous polypropylene mesh. Because it allows a greater flow of cells through the pores in this phase of prosthesis incorporation, which in turn contributes to a better collagen deposition and maturation at a later stage as the intensity of inflammation gets lower. However, the maintenance of the inflammatory process for a long time, as occurs with tissue separating meshes with absorbable components, determines a lesser collagen deposition.

The present study, on the 21st postoperative day, conducted an evaluation in a single moment with three different types of lightweight and macroporous meshes. It is questioned whether at a later time, as *Maeda et al*.^20^ described, this process of tissue inflammation could reduce and fibroplasia could improve, respectively, with the reduction of the inflammatory cellularity and maturation of the deposited collagen, which is consistent with the foreign body reaction that characterizes the implantation of synthetic meshes in the repair of hernias and abdominal wall defects.

Gruber-Blum *et al*.^23^ carried out an experimental study in which the authors implanted a macroporous and medium-weight polypropylene mesh intraperitoneally in rats with or without protection using one of three different types of anti-adhesive barrier fixed to the mesh with a fibrin sealant. Histological analysis showed that healing process, foreign body reaction, and neovascularization occurred in all groups. The tissue ingrowth of the mesh was impaired in groups with cover based on porcine collagen and carboxymethylcellulose, but in the group with a polylactic acid-based barrier and in the unprotected control there was good tissue integration of the mesh. The present study acquired a similar finding. Groups with polypropylene/polyglecaprone and polyester/porcine collagen meshes showed a less advanced and immature incorporation process compared to the tissue repair process of the sham group, although with no significant differences to polypropylene mesh group, which presented a tissue maturation process similar as the one of the sham group. Therefore, although the anti-adhesive barriers can reduce the formation of adhesions between the visceral surface and the intra-abdominal organs and structures of the mesh, they may potentially impair tissue ingrowth and incorporation of the polypropylene mesh parietal face to the abdominal wall.

The groups with meshes showed a more intense and significant inflammatory response on the mesh surface compared to that of the sham group, especially the groups with meshes made of polypropylene/polyglecaprone and polyester/porcine collagen. This difference was evidenced by the more abundant number of macrophages and giant cells in the groups with meshes, which characterize a well-defined phase of the foreign body reaction. Although there were no statistically significant differences between the three groups with meshes, there was a tendency for a greater number of macrophages and gigantocytes in groups with tissue separating meshes than in the polypropylene mesh group. The presence of a high number of gigantocytes in the polypropylene/polyglecaprone mesh group characterized a more intense foreign body reaction, probably associated with the degradation process of the polyglecaprone used in making the anti-adhesive barrier.

Despite the 21 postoperative days, large fragments of the polyglecaprone barriers remained, surrounded by clusters of macrophages and giant foreign body cells. The use of synthetic meshes for the repair of defects in the abdominal wall, especially tissue separating meshes, implies a greater amount of degradable synthetic or biological polymers that determine an inflammatory response accompanied by a greater number of macrophages M1 and M2 and giant cells capable of leading to the biodegradation of this absorbable material and the involvement of the nonabsorbable filaments of the mesh, during the process of fibroplasia, incorporating the mesh to the tissue^24^.

Pascual *et al*.^23^ carried out an experimental study with rabbits to evaluate the changes that different types of polypropylene meshes cause on growth factors and the recruitment of macrophages during the early phase of mesh incorporation. The authors used four different types of polypropylene meshes, which differed in density (light or heavy) and porosity (micro and macroporous) and which were partially absorbable in association with polyglecaprone. Regarding the inflammatory response, the authors have noticed the presence of many inflammatory cells, macrophages and giant foreign body cells in the vicinity of mesh filaments in all groups. In the polypropylene with polyglecaprone mesh group, there was a greater number of giant foreign body cells surrounding absorbable filaments, as well as a higher percentage of macrophages, compared to the other groups. However, there was no significant difference in macrophage count in the meshes composed exclusively of polypropylene. However, the results of the present study and those of Gruber-Blum *et al*.^24^ did not show significant differences between groups with or without an anti-adhesive barrier in relation to the intensity of the foreign body reaction, although there was a greater tendency to it in the polypropylene/polyglecaprone mesh group.

The sham group had the lowest total inflammation score compared to the groups with polypropylene, polypropylene/polyglecaprone, and polyester/porcine collagen meshes. There were no statistically significant differences between the groups with meshes, although there was a tendency towards higher values in inflammation scores in the polypropylene/polyglecaprone and polyester/porcine collagen meshes groups compared to the polypropylene mesh group. This result was similar to the ones by Ultrabo et *al*.^25^, who did not find statistically significant differences in the inflammatory reaction score after 30 days of preperitoneal implantation in rats with micro and macroporous polypropylene and macroporous polypropylene with poliglecaprone meshes. The presence of a synthetic mesh with or without a tissue separating barriers implies a more intense and prolonged inflammatory response than the simple suture of the median aponeurosis of the abdominal wall using a polyglactin suture. This finding is compatible with the greater amount of synthetic or biological material needed to make the meshes, particularly the tissue separating meshes that have at least two faces^13^.

Maeda *et al*.^20^ carried out an experimental study with Wistar rats involving the creation of a hernial defect in the abdominal wall and the implantation of four different types of meshes in a preperitoneal position: high-density polypropylene, low-density polypropylene, polypropylene encapsulated with polydioxanone covered with oxidized cellulose, and expanded polytetrafluoroethylene (PTFE-e). The score for late inflammatory response (28 days postoperatively) was lower in the light polypropylene group compared to the other ones, as well as in the heavy polypropylene group compared to the polypropylene with cellulose group.

Fuziy *et al*.^21^ carried out an experimental study with rats and implanted one of four different types of meshes intraperitoneally. The meshes were made of PTFE-e, polypropylene with oxidized cellulose, polypropylene with silicone, and only polypropylene. Regarding the inflammation score, the PTFE-e mesh group had a higher and statistically significant score compared to all other groups. In contrast, the group of polypropylene meshes with silicone showed the lowest inflammation score and had a statistical significance in comparison to polypropylene meshes with oxidized cellulose and PTFE-e, but with no significant difference to meshes made exclusively of polypropylene. However, in the present study, there were no significant differences in relation to the total inflammation score between the groups with meshes, although the polypropylene mesh group tended to show lower values compared to the groups with anti-adhesive polyglecaprone or porcine collagen meshes.

The sham group showed a higher and significant tissue proportion of type I/III collagen compared to the groups with polypropylene/polyglecaprone and polyester/porcine collagen meshes, although there was no significant difference in relation to the polypropylene mesh group, which stood out among the groups with meshes as the group with the highest proportion of type I/III collagen between the mesh and the host tissue. The sham group was characterized by the presence of type I collagen fibers organized in a more aligned, uniform way and grouped in dense birefringent bundles in comparison to the other groups. It denotes a more advanced fibroplasia and collagen maturation process of the sham group compared to the groups with meshes. Collagen maturation in the polypropylene mesh group was similar as that of the sham group in the tissue proportion of type I/III collagen and collagen maturation. These findings suggest that the presence of a tissue separating barriers based on polyglecaprone and/or porcine collagen meshes may negatively affect the homeostasis of mature collagen (type I collagen) in the newly formed extracellular matrix.

The morphometric analysis by Maeda *et al*.^20^, previously described, demonstrated that the PTFE-e group showed a higher amount of collagen on the 7th postoperative day in relation to the groups with high-density polypropylene, low-density polypropylene, and polypropylene encapsulated with polydioxanone covered with oxidized cellulose, and that the group of polypropylene with cellulose showed a higher amount of collagen compared to the group of heavy polypropylene. However, the late morphometric analysis, performed on the 28th postoperative day, did not show any significant differences between the groups. Similarly, in the present study, despite a tendency towards a higher tissue proportion of type I/III collagen in the polypropylene mesh group, there were no significant differences between the polypropylene mesh group compared to the polypropylene/polyglecaprone and polyester/porcine collagen meshes.

Biondo-Simões *et al*.^26^ carried out a comparative experimental study in Wistar rats with pre-peritoneal implants using two different types of mesh for correction of defects induced in the abdominal wall with maintenance of the integrity of the parietal peritoneum. They used a heavy-weight polypropylene mesh and a partially absorbable mesh composed of lightweight polypropylene with polyglecaprone. The quantity and quality of collagen were evaluated at five different time intervals using picrosirius red staining. Regarding fibroplasia, there was a gradual and progressive gain in both groups of mesh in relation to the total amount of collagen, without significant differences between the groups, although in the first two weeks there was a predominance of type III collagen deposition. After this observation period, the amount of type I collagen increased steadily and progressively, surpassing the amount of type III collagen, which stabilized. Although collagen deposition was slightly higher in the polypropylene mesh group at all times, there were no significant differences. There was an irregular disposition of collagen fibers in the first weeks, followed by the deposition of thicker fibers with a regular disposition.

The present study showed such irregularity in the disposition of collagen fibers in groups with meshes, especially in the groups with polypropylene/polyglecaprone and polyester/porcine collagen meshes in comparison to the sham group. It denotes a delay in the fibroplasia phase and in the maturation of collagen in the extracellular matrix in groups with tissue separating meshes.

The stratification of inflammation scores into three categories (mild, moderate, and intense) allowed identifying that there is an inversely proportional correlation between inflammation scores and the proportion of type I/III collagen between the mesh and the host tissue. Although the inflammatory response is necessary for healing process, tissue repair and incorporation of synthetic meshes, a marked and prolonged inflammatory response may compromise the subsequent fibroplasia phase and affect the deposition of various types of collagen in the newly formed extracellular matrix, thus determining a delay in the stage of collagen maturation characterized by lower proportions of type I/III collagen^4^^,^ 10 ^,^ 15 ^,^ 27.

The sham group had the lowest inflammation score and the best proportion of type I/III collagen in relation to groups with tissue separating meshes. Although there was a significant difference between the sham group and the group with polypropylene mesh as for the inflammation score, there was no significant difference in relation to the proportion of type I/III collagen. However, the aspect of the type I collagen fiber arrangement in the sham group shows a more organized stage of fibroplasia in comparison to all groups with meshes. Therefore, the greater and more prolonged the inflammatory response between the mesh and the host tissue, the lower the proportion of type I/III collagen, and the worse the fibroplasia response and collagen maturation in the extracellular matrix^15^^,^ 16 ^,^ 20. This trend occurred in groups with tissue separating meshes compared to the polypropylene mesh group, particularly the sham group.

The use of experimental models to assess biocompatibility, inflammatory response, local fibroplasia, foreign body reaction, mesh-tissue interaction, and changes in biomechanical properties of synthetic fabrics with or without an anti-adhesive barrier mechanism is impractical in humans for ethical reasons. Therefore, it can only be evaluated in studies with animals^28^.

There is a great variability as for the polymers that make up the meshes (porosity, weight, pore shape, biomechanical properties, and fixation), and type of implant, models of defects induced in the abdominal wall, types of animal, types of inflammation, and fibroplasia scores intended for assessing the mesh incorporation process^27^^,^ 28. This implies technical limitations for comparing experimental studies using meshes to repair induced abdominal wall defects^27^^,^ 29 ^,^ 30. Such heterogeneity in the design of studies and the impossibility of carrying them out in full in human beings impose a limitation to the extrapolation of results and conclusions to the clinical scenario. However, experimental studies may contribute to the development of synthetic or biological meshes with greater biocompatibility, less tissue inflammatory reactions, foreign body reaction, better fibroplasia, and respect for the biomechanical properties of the implanted mesh and the abdominal wall, which offer a better performance of the mesh and interaction tissue-mesh^8^.

## Conclusions

The intraperitoneal implantation of meshes, especially tissue separating meshes made of polypropylene/polyglecaprone and polyester/porcine collagen, determined a more intense and longer tissue inflammatory response compared to repairs with polyglactin suture in the musculoaponeurotic plane. As well as, the groups with meshes presented a more immature and disorganized fibroplasia, marked by a reduction in the tissue proportion of type I/III collagen, and a greater foreign body reaction. However without significant differences between the polypropylene mesh group and the groups with tissue separating meshes.
